# How Well Do We Know Wellens?

**DOI:** 10.7759/cureus.27841

**Published:** 2022-08-10

**Authors:** Riya Sam, Zain Jafri, Muftawu-Deen Iddrisu, Dil Patel

**Affiliations:** 1 Internal Medicine, Advocate Illinois Masonic Medical Center, Chicago, USA; 2 Internal Medicine, Advocate Illinois Masonic Medical Center, Chicago , USA; 3 Internal Medicine, Rosalind Franklin University of Medicine and Science, North Chicago, USA

**Keywords:** ekg abnormalities, atypical chest pain, lad occlusion, acute st elevation myocardial infarction, wellens

## Abstract

Wellens' syndrome is a pattern of electrocardiographic T-wave changes that is associated with critical left anterior descending artery (LAD) stenosis. This syndrome continues to be under-recognized by clinicians and carries a significant risk of mortality if not intervened timely. We describe the case of an elderly Chinese woman who initially presented to the outpatient clinic with atypical chest pain. A routine EKG obtained in the office was documented as non-ischemic and was sent for a dobutamine stress echocardiogram. Pretest two-dimensional (2D) echocardiogram demonstrated akinesis and aneurysmal deformity of the entire apical myocardium and upon review of the previous EKG, Type 1 Wellens' sign was noted prompting emergent coronary angiogram, which revealed critical LAD stenosis (99%). She underwent successful percutaneous coronary intervention (PCI) with drug-eluting stents.

## Introduction

Alternatively known as anterior descending T-wave syndrome, Wellens' syndrome was first reported in 1982 by de Zwaan et al. in a subgroup of patients with unstable angina during a pain-free period [1]. Seven years later, the same authors performed another prospective study on patients with Wellens' syndrome and confirmed 100% association with a significant proximal left anterior descending artery (LAD) disease by cardiac coronary angiography. It describes a pattern of electrocardiographic changes, particularly deeply inverted or biphasic T-waves in leads V2-V3, which is highly specific for critical, proximal stenosis of the LAD. In clinical practice, stress testing might be performed due to the lack of typical ST segment elevation in Wellens’ syndrome to obtain more evidence of cardiac ischemia. However, increasing cardiac demand during stress testing may result in acute myocardial infarction, and even fatal dysrhythmia and death. Therefore, it is essential to consider the coronary angiogram as the initial diagnostic modality instead of other conservative examinations in patients with EKG patterns, indicating a possibility of Wellens’ syndrome. 

## Case presentation

**Figure 1:**
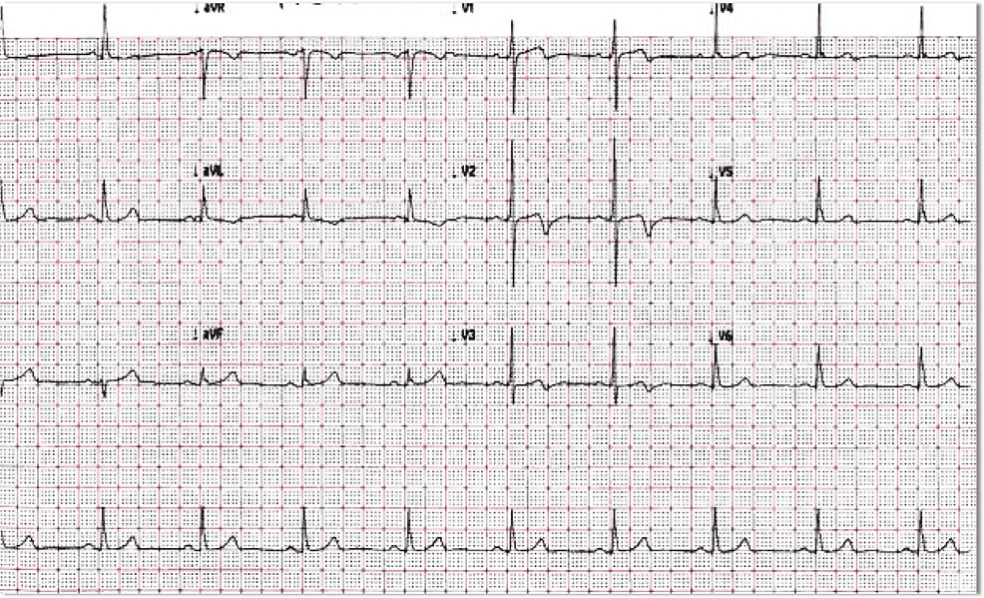
Initial EKG with biphasic T-waves in V2 and V3

A 77-year-old Chinese woman with a past medical history of hypertension presented to her primary care physician (PCP) for a week-long history of shortness of breath and exertional chest pain that radiated up to the base of her throat. She had tried unsuccessfully using aspirin and Chinese herbs to alleviate her pain. A comprehensive cardiovascular examination was unremarkable. An EKG obtained was documented as normal sinus rhythm with nonspecific T-wave changes. She was instructed to continue the aspirin and follow up with an echocardiogram stress test. Pretest echocardiogram prior to dobutamine stress echo done a month later revealed akinesis and aneurysmal deformity of the entire apical myocardium and akinesis of the mid-apical anteroseptal myocardium, thus aborting the stress test. A review of her medical records revealed that there were biphasic T-waves evident in anterior leads concerning Wellens' syndrome, which was overlooked by her PCP ( Figure 1). 

**Figure 2:**
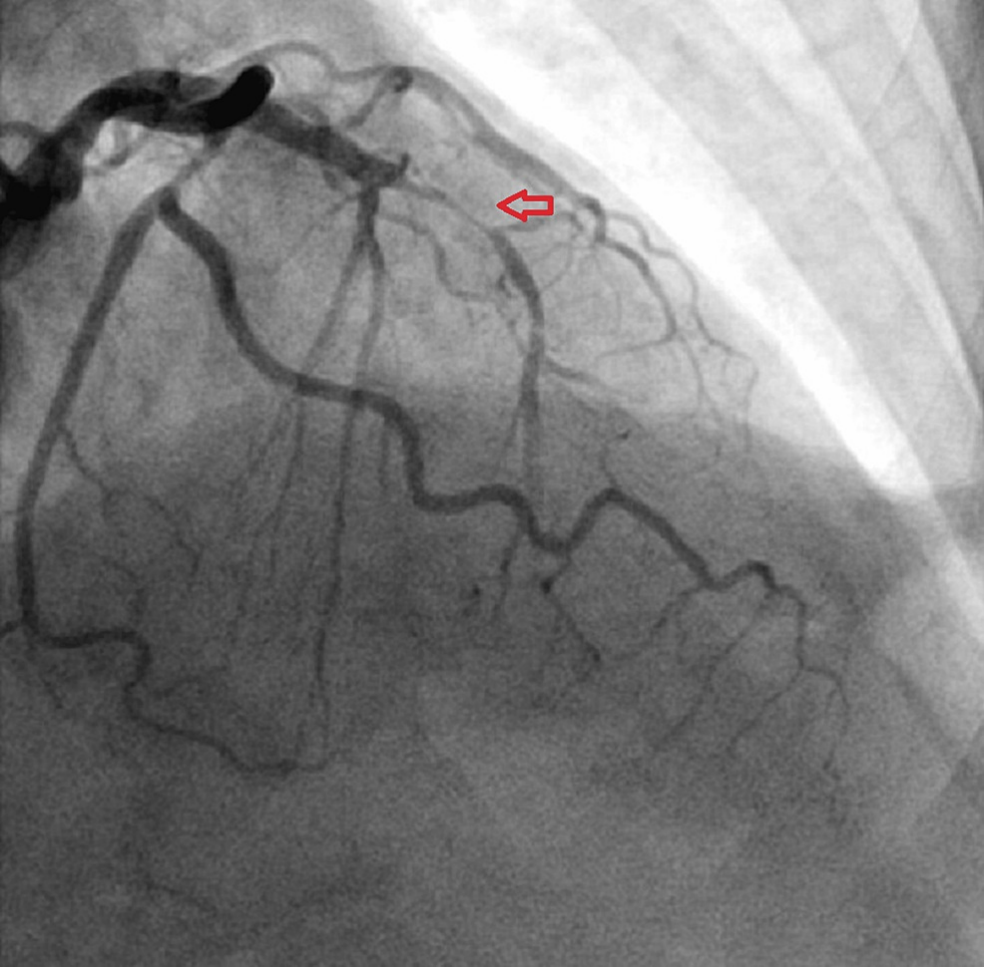
Coronary angiogram demonstrating critical occlusion of mid-LAD LAD: left anterior descending artery

**Figure 3:**
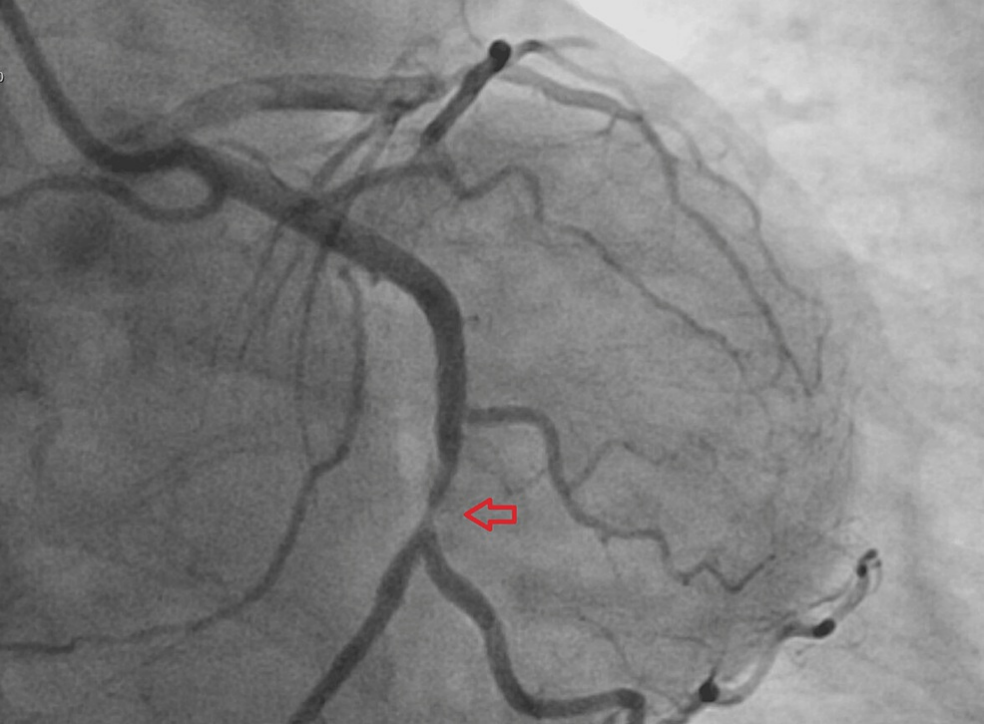
Coronary angiogram demonstrating occlusion of LCX LCX: left circumflex artery

**Figure 4:**
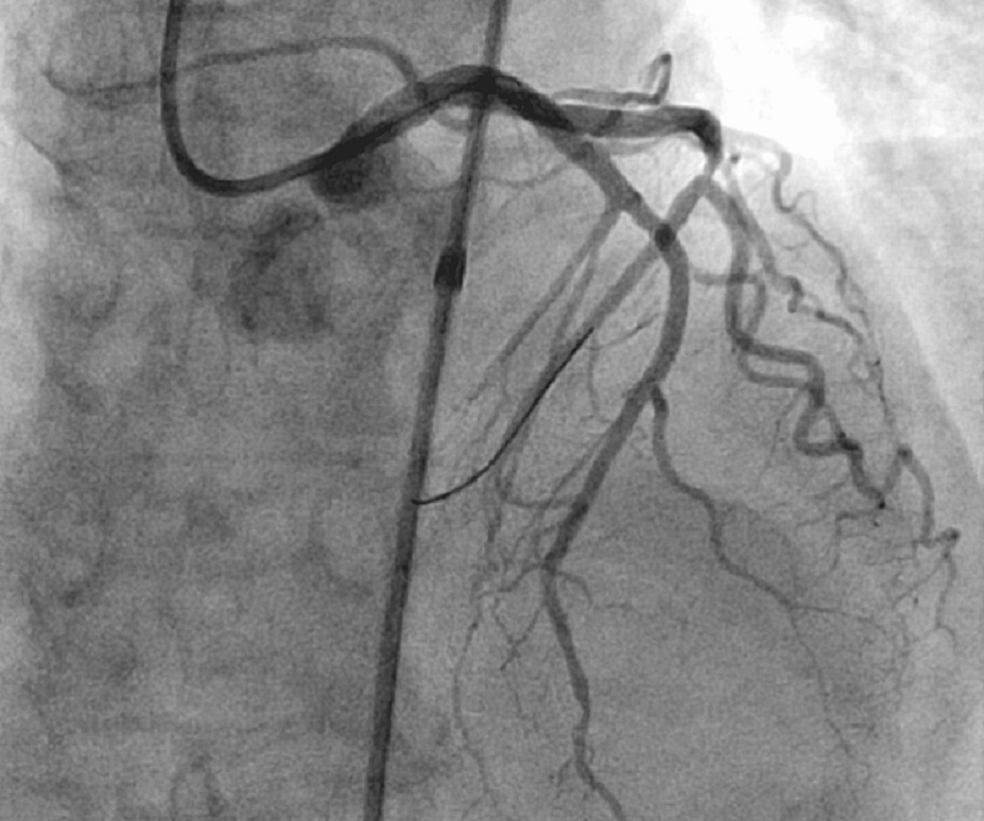
Coronary angiogram demonstrating complete revascularization of LAD and LCX after placement of drug-eluting stents LAD: left anterior descending artery; LCX: left circumflex artery

She was immediately transferred to the emergency department and admitted for emergent cardiac catheterization. Vitals were remarkable for a heart rate of 60 beats per minute. Labs were remarkable for troponin: 2.15 (Reference range: <=0.04 ng/mL). The EKG was notable for biphasic T-wave patterns in leads V1 - V4. She was loaded with full dose aspirin and underwent an emergent coronary angiogram, which confirmed severe stenosis of mid LAD (99%) (Figure 2) and left circumflex artery (Figure 3). Successful percutaneous transluminal coronary angioplasty with drug-eluting stents of the mid-LAD and mid-circumflex artery was performed (Figure 4). She was started on metoprolol, lisinopril, high-dose atorvastatin, and dual antiplatelet therapy with low-dose aspirin and clopidogrel. She was discharged home the next day with instructions to follow up with cardiology. 

## Discussion

Wellens’ syndrome is described as an abnormal EKG pattern consisting of deep T-wave inversions in the anterior chest leads associated with critical, proximal stenosis of the LAD.  It consists of a clinical, laboratory, and EKG criterion, which involves a prior history of angina, minimal or no elevation of cardiac enzymes, minimal or no elevation of ST segment (<1mm), no pathological precordial Q waves, preservation of precordial R-wave progression and finally biphasic T-waves or deeply inverted T-waves present in leads V2 and V3.  Lastly, it is important to note that the EKG is performed in a pain-free state as in symptomatic patients T-wave abnormalities appear to normalize or progress to ST-segment elevation [1,2] . In 1982 and 1989, de Zwaan and his colleagues first described it in their two original studies. The characteristic EKG patterns of  the syndrome were present in 26 out of 145 (18%), and in 180 out of 1,260 (14%) patients who were admitted for unstable angina in the first and second original study, respectively [3-5]. They also noted that 100% of these patients had LAD lesions, with subsequent development of extensive anterior wall acute myocardial infarctions within weeks for 75% of patients who did not receive coronary revascularization [3-5].  

With a reported incidence of about 15% of all acute coronary syndromes in the United States, this syndrome is considered a pre-infarction stage of coronary artery disease (CAD). Typical risk factors include hypertension, diabetes mellitus, family history of premature heart disease, smoking, hyperlipidemia,  metabolic syndrome, and occupational stress [4,5]. Some cases have also been reported in patients without cardiovascular risk factors. Wellens’ syndrome is classified into two main types. Type 1 is found in roughly 24% of cases and has a biphasic T-wave, with initial positivity and terminal negativity. Type 2 has deeply and symmetrically inverted T-waves and is seen in 76% of cases [6,7]. Even though it is highly specific for proximal LAD stenosis, there exist mimics of Wellens syndrome or pseudo-Wellens syndrome, including congenital myocardial bridge, stress cardiomyopathy, and substance abuse [3,5]. Other notable causes of T-wave inversions are central nervous system injury (so-called “cerebral” T-waves), left ventricular hypertrophy, pulmonary embolism, right bundle branch block , and hypertrophic cardiomyopathy [6].  Hence, clinicians need to be able to distinguish between Wellens’ syndrome and pseudo-Wellens. An urgent coronary angiogram is recommended whenever Wellens' syndrome is suspected. Majority of patients who do not receive coronary revascularization develop subsequent anterior wall myocardial infarctions despite symptomatic treatment. As such patients have critical narrowing of the LAD, a stress test can provoke an acute myocardial infarction and should be avoided.

Despite being well described, clinicians and PCPs tend to overlook this characteristic EKG finding as described in this case. Additionally, our case is unique with respect to the location of the LAD lesion, which is typically proximal and highlights the significance of prompt recognition of Wellens' syndrome on an outpatient evaluation of "atypical chest pain" and urgent referral for intervention and management.

## Conclusions

To conclude, having an in-depth understanding of Wellens’ syndrome would allow physicians to diagnose and treat impending anterior myocardial infarctions. Moreover, it would also prevent misreading the EKG changes as “nonspecific,” especially, when those patients are at risk of being further investigated through stress imaging, placing them at an even greater risk of adverse events and fatal outcomes. Therefore, timely recognition of Wellens’ syndrome can make the difference between life and death. 
